# Supplementary material for: Prevalence of pectus excavatum in an adult population-based cohort estimated from radiographic indices of chest wall shape
Source: PLoS One. 2020 May 7;15(5):e0232575. doi: 10.1371/journal.pone.0232575 (PMC7205298; doi:10.1371/journal.pone.0232575)
Supplement: S1 Table — (DOCX) [file pone.0232575.s001.docx]

**Supplementary Table 1. Demographic Characteristics of Participants**

|  | **Pectus Cases**  **(n=297)** | | **DHS1**  **(n=2687)** | | **DHS2**  **(Entire group)**  **(n=1780)** | | **DHS2**  **(Not in DHS1)**  **(n=788)** | | **DH2 (Repeated Evaluation; Subset of DHS1)**  **(n=992)** | |
| --- | --- | --- | --- | --- | --- | --- | --- | --- | --- | --- |
|  | **N** | **Summary** | **N** | **Summary** | **N** | **Summary** | **N** | **Summary** | **N** | **Summary** |
| Male (%) | 297 | 231 (78) | 2687 | 1158 (43) | 1780 | 527 (30) | 788 | 249 (32) | 992 | 278 (28) |
| Ethnicity |  |  |  |  |  |  |  |  |  |  |
| Non-Hispanic White, N (%) | 297 | 270 (91) | 2687 | 824 (31) | 1780 | 980 (55) | 788 | 218 (28) | 992 | 319 (32) |
| Non-Hispanic Black, N (%) | 297 | 2 (1) | 2687 | 1342 (50) | 1780 | 537 (30) | 788 | 450 (57) | 992 | 530 (53) |
| Hispanic, N (%) | 297 | 10 (3) | 2687 | 467 (17) | 1780 | 220 (12) | 788 | 102 (13) | 992 | 118 (12) |
| Other, N (%) | 297 | 15 (5) | 2687 | 54 (2) | 1780 | 43 (2) | 788 | 18 (2) | 992 | 25 (3) |
| Age |  |  |  |  |  |  |  |  |  |  |
| Years, median (IQR) | 297 | 15 (14-47) | 2687 | 44 (37-52) | 1780 | 50 (42-58) | 788 | 47 (37-57) | 992 | 50 (42-58) |
| Years, mean, SD | 297 | 16.1 ± 4.0 | 2687 | 45.0 ± 9.3 | 1780 | 50.0 ± 10.6 | 788 | 47.0 ± 11.7 | 992 | 52.1 ± 9.1 |
| Years, median (min-max) | 297 | 15 (10–52) | 2687 | 44 (29-67) | 1780 | 50 (18-85) | 788 | 47 (18-85) | 992 | 50 (18-85) |

Abbreviations: DHS1, Dallas Heart Study 1; DHS2, Dallas Heart Study 2; IQR, interquartile range; SD, standard deviation; min, minimum; max, maximum
