# Supplementary material for: Prevalence of pectus excavatum in an adult population-based cohort estimated from radiographic indices of chest wall shape
Source: PLoS One. 2020 May 7;15(5):e0232575. doi: 10.1371/journal.pone.0232575 (PMC7205298; doi:10.1371/journal.pone.0232575)
Supplement: S2 Table — (DOCX) [file pone.0232575.s002.docx]

**Supplementary Table 2. Haller and Correction Index Measurements in Pectus Cases and DHS Cohorts at Multiple Axial Levels (T6, T8 and Superior Xiphoid) by Gender**

|  |  | **All** | | **Male** | | **Female** | | **Male vs Female** |
| --- | --- | --- | --- | --- | --- | --- | --- | --- |
| **Population** | **Pectus Measurement** | **N** | **Median (IQR)** | **N** | **Median (IQR)** | **N** | **Median (IQR)** | **P-value** |
| Pectus Cases | Haller Index at T6 | 297 | 3.60 (3.04-4.16) | 231 | 3.54 (3.00-4.06) | 66 | 3.84 (3.20-4.42) | **0.019** |
|  | Haller Index at T8 | 297 | 3.44 (2.90-4.12) | 231 | 3.40 (2.89-3.99) | 66 | 3.76 (3.02-4.45) | **0.032** |
|  | Haller Index at Superior Xiphoid | 297 | 3.93 (3.19-4.67) | 231 | 3.79 (3.17-4.54) | 66 | 4.25 (3.43-5.25) | **0.011** |
|  | Haller Index at Max Depression | 278 | 4.04 (3.34-4.75) | 215 | 3.90 (3.29-4.62) | 63 | 4.38 (3.75-5.52) | **0.0090** |
|  | Correction Index at T6 | 297 | 10 (5-16) | 231 | 9 (5-15) | 66 | 14 (9-21) | **<0.001** |
|  | Correction Index at T8 | 297 | 18 (11-27) | 231 | 17 (10-25) | 66 | 23 (14-34) | **0.0041** |
|  | Correction Index at Superior Xiphoid | 297 | 29 (21-39) | 231 | 29 (20-38) | 66 | 32 (26-43) | **0.018** |
|  | Correction Index at Max Depression | 278 | 30 (23-40) | 215 | 29 (22-38) | 63 | 34 (26-44) | **0.0090** |
| DHS1 | Haller Index at T6 | 2687 | 2.19 (2.02-2.38) | 1158 | 2.18 (2.01-2.36) | 1529 | 2.20 (2.03-2.40) | **0.016** |
|  | Haller Index at T8 | 2687 | 2.15 (1.99-2.35) | 1158 | 2.14 (1.97-2.31) | 1529 | 2.17 (2.00-2.38) | **<0.0001** |
|  | Haller Index at Superior Xiphoid | 2687 | 2.14 (1.98-2.33) | 1158 | 2.12 (1.97-2.29) | 1529 | 2.17 (1.98-2.35) | **<0.001** |
|  | Correction Index at T6 | 2687 | 3.9 (1.9-6.3) | 1158 | 3.2 (1.4-5.4) | 1529 | 4.4 (2.3-7.0) | **<0.0001** |
|  | Correction Index at T8 | 2687 | 4.1 (1.9-6.5) | 1158 | 3.7 (1.5-5.8) | 1529 | 4.5 (2.1-7.0) | **<0.0001** |
|  | Correction Index at Superior Xiphoid | 2687 | 4.2 (2.2-6.4) | 1158 | 3.7 (1.7-5.8) | 1529 | 4.6 (2.4-7.0) | **<0.0001** |
| DHS2 | Haller Index at T6 | 788 | 2.23 (2.04-2.44) | 249 | 2.16 (2.00-2.34) | 539 | 2.25 (2.06-2.47) | **<0.0001** |
| (Not in DHS1) | Haller Index at T8 | 788 | 2.16 (1.98-2.38) | 249 | 2.07 (1.94-2.25) | 539 | 2.21 (2.02-2.41) | **<0.0001** |
|  | Haller Index at Superior Xiphoid | 788 | 2.16 (1.97-2.37) | 249 | 2.08 (1.93-2.25) | 539 | 2.20 (2.01-2.40) | **<0.0001** |
|  | Correction Index at T6 | 788 | 3.9 (1.8-6.3) | 249 | 3.1 (1.5-5.5) | 539 | 4.3 (2.1-6.8) | **<0.0001** |
|  | Correction Index at T8 | 788 | 3.2 (1.0-5.9) | 249 | 2.1 (0.5-4.8) | 539 | 3.5 (1.4-6.6) | **<0.0001** |
|  | Correction Index at Superior Xiphoid | 788 | 3.4 (1.3-5.9) | 249 | 2.6 (0.8-4.6) | 539 | 3.6 (1.6-6.5) | **<0.0001** |
| DHS1 | Haller Index at T6 | 992 | 2.23 (2.0-2.44) | 278 | 2.19 (2.05-2.38) | 714 | 2.25 (2.06-2.45) | **0.015** |
| (Repeated | Haller Index at T8 | 992 | 2.20 (2.04-2.41) | 278 | 2.16 (2.00-2.34) | 714 | 2.22 (2.05-2.44) | **<0.001** |
| In DHS2) | Haller Index at Superior Xiphoid | 992 | 2.19 (2.01-2.40) | 278 | 2.15 (1.99-2.33) | 714 | 2.21 (2.02-2.42) | **0.029** |
|  | Correction Index at T6 | 992 | 4.0 (2.0-6.5) | 278 | 2.9 (1.1-5.1) | 714 | 4.5 (2.3-7.0) | **<0.0001** |
|  | Correction Index at T8 | 992 | 4.6 (2.0-7.0) | 278 | 3.5 (1.1-5.8) | 714 | 5.0 (2.4-7.4) | **<0.0001** |
|  | Correction Index at Superior Xiphoid | 992 | 4.5 (2.2-6.7) | 278 | 3.5 (1.5-5.6) | 714 | 4.8 (2.5-7.4) | **<0.0001** |
| DHS2 | Haller Index at T6 | 992 | 2.22 (2.03-2.43) | 278 | 2.21 (2.04-2.37) | 714 | 2.23 (2.03-2.45) | 0.28 |
| (Repeat | Haller Index at T8 | 992 | 2.17 (1.99-2.37) | 278 | 2.14 (1.98-2.30) | 714 | 2.18 (2.00-2.38) | **0.023** |
| From DHS1) | Haller Index at Superior Xiphoid | 992 | 2.15 (1.99-2.35) | 278 | 2.13 (1.97-2.30) | 714 | 2.17 (1.99-2.37) | **0.039** |
|  | Correction Index at T6 | 992 | 3.8 (1.8-6.3) | 278 | 3.2 (1.5-5.6) | 714 | 4.1 (1.9-6.7) | **0.00014** |
|  | Correction Index at T8 | 992 | 3.4 (1.3-5.9) | 278 | 3.0 (1.4-5.0) | 714 | 3.6 (1.2-6.3) | **0.024** |
|  | Correction Index at Superior Xiphoid | 992 | 3.6 (1.7-6.1) | 278 | 3.0 (1.6-5.0) | 714 | 3.9 (1.8-6.6) | **0.00061** |

P-values calculated using the Wilcoxon rank sum test. Abbreviations: DHS1, Dallas Heart Study 1; DHS2, Dallas Heart Study 2; IQR, interquartile range
