# Supplementary material for: Prevalence of pectus excavatum in an adult population-based cohort estimated from radiographic indices of chest wall shape
Source: PLoS One. 2020 May 7;15(5):e0232575. doi: 10.1371/journal.pone.0232575 (PMC7205298; doi:10.1371/journal.pone.0232575)
Supplement: S3 Table — (DOCX) [file pone.0232575.s003.docx]

**Supplementary Table 3. Prevalence of Pectus Excavatum in Cases and Population-based Cohorts**

|  |  | **Pectus Cases** | | **Male** | | **Female** | |
| --- | --- | --- | --- | --- | --- | --- | --- |
| **Population** | **Pectus Definition** | **N** | **N (%)** | **N** | **N (%)** | **N** | **N (%)** |
| Pectus Cases | HI>3.25, T6 | 297 | 189 (64) | 231 | 141 (61) | 66 | 48 (73) |
|  | HI>3.25, T8 | 297 | 176 (59) | 231 | 133 (58) | 66 | 43 (65) |
|  | HI>3.25, Max Depression | 278 | 218 (78) | 215 | 166 (77) | 63 | 52 (82) |
|  | CI>10%, T6 | 297 | 150 (50) | 231 | 104 (45) | 66 | 46 (70) |
|  | CI>10%, T8 | 297 | 229 (77) | 231 | 174 (75) | 66 | 55 (83) |
|  | CI>10%, Max Depression | 278 | 265 (95) | 215 | 205 (95) | 63 | 60 (95) |
|  | HI>3.25 and CI>10%, T6 | 297 | 125 (42) | 231 | 86 (37) | 66 | 39 (59) |
|  | HI>3.25 and CI>10%, T8 | 297 | 165 (56) | 231 | 123 (53) | 66 | 42 (64) |
| DHS1 | HI>3.25, T6 | 2687 | 10 (0.4) | 1158 | 3 (0.3) | 1529 | 7 (0.5) |
|  | HI>3.25, T8 | 2687 | 11 (0.4) | 1158 | 4 (0.3) | 1529 | 7 (0.5) |
|  | CI>10%, T6 | 2687 | 155 (6) | 1158 | 44 (4) | 1529 | 111 (7) |
|  | CI>10%, T8 | 2687 | 175 (6) | 1158 | 46 (4) | 1529 | 129 (8) |
|  | HI>3.25 and CI>10%, T6 | 2687 | 4 (0.1) | 1158 | 0 (0) | 1529 | 4 (0.3) |
|  | HI>3.25 and CI>10%, T8 | 2687 | 10 (0.4) | 1158 | 3 (0.3) | 1529 | 7 (0.5) |
| DHS2 | HI>3.25, T6 | 788 | 7 (0.9) | 249 | 0 (0) | 539 | 7 (1) |
| (Not in DHS1) | HI>3.25, T8 | 788 | 5 (0.6) | 249 | 0 (0) | 539 | 5 (1) |
|  | CI>10%, T6 | 788 | 54 (7) | 249 | 5 (2) | 539 | 49 (9) |
|  | CI>10%, T8 | 788 | 52 (7) | 249 | 5 (2) | 539 | 47 (9) |
|  | HI>3.25 and CI>10%, T6 | 788 | 2 (0.3) | 249 | 0 (0) | 539 | 2 (0.4) |
|  | HI>3.25 and CI>10%, T8 | 788 | 3 (0.4) | 249 | 0 (0) | 539 | 3 (0.6) |
| DHS2 | HI>3.25, T6 | 992 | 9 (0.9) | 278 | 1 (0.4) | 714 | 8 (1.1) |
| (Subset of DHS1) | HI>3.25, T8 | 992 | 8 (0.8) | 278 | 1 (0.4) | 714 | 7 (1) |
|  | CI>10%, T6 | 992 | 73 (7.4) | 278 | 7 (2.5) | 714 | 66 (9.2) |
|  | CI>10%, T8 | 992 | 71 (7.2) | 278 | 9 (3.2) | 714 | 62 (8.7) |
|  | HI>3.25 and CI>10%, T6 | 992 | 5 (0.5) | 278 | 1 (0.4) | 714 | 4 (0.6) |
|  | HI>3.25 and CI>10%, T8 | 992 | 8 (0.8) | 278 | 1 (0.4) | 714 | 7 (1) |

P-values calculated using Fisher exact test.

Abbreviations: DHS1, Dallas Heart Study 1; DHS2, Dallas Heart Study 2; HI, Haller Index; CI, Correction Index
