# Supplementary material for: Prevalence of pectus excavatum in an adult population-based cohort estimated from radiographic indices of chest wall shape
Source: PLoS One. 2020 May 7;15(5):e0232575. doi: 10.1371/journal.pone.0232575 (PMC7205298; doi:10.1371/journal.pone.0232575)
Supplement: S4 Table — (DOCX) [file pone.0232575.s004.docx]

**Supplementary Table 4. Chest Wall Shapes in Population-based Cohorts by Ethnicity**

|  |  |  | **Median (IQR)** | | | | **p-values** | | |
| --- | --- | --- | --- | --- | --- | --- | --- | --- | --- |
| **Cohort** | **Pectus Measurement** | **N** | **Black** | **White** | **Hispanic** | **Other** | **Global** | **Black vs White** | **Black vs Hispanic** |
| DHS1 | Haller Index at T6 | 2687 | 2.18 (2.03-2.37) | 2.22 (2.03-2.42) | 2.13 (1.99-2.34) | 2.32 (2.13-2.56) | **<0.0001** | 0.052 | **<0.001** |
|  | Haller Index at T8 | 2687 | 2.15 (2.00-2.35) | 2.18 (2.01-2.38) | 2.10 (1.93-2.26) | 2.33 (2.06-2.52) | **<0.0001** | **0.013** | **<0.0001** |
|  | Haller Index at Sup. Xiphoid | 2687 | 2.14 (1.98-2.32) | 2.17 (2.00-2.36) | 2.09 (1.93-2.24) | 2.28 (2.06-2.52) | **<0.0001** | **0.0062** | **<0.0001** |
|  | Correction Index at T6 | 2687 | 3.4 (1.6-5.7) | 4.2 (2.1-7.0) | 4.4 (2.5-6.9) | 3.5 (1.6-6.0) | **<0.0001** | **<0.0001** | **<0.0001** |
|  | Correction Index at T8 | 2687 | 3.6 (1.4-5.8) | 4.9 (2.3-7.5) | 4.4 (2.4-6.8) | 4.2 (2.5-6.9) | **<0.0001** | **<0.0001** | **<0.0001** |
|  | Correction Index at Sup. Xiphoid | 2687 | 3.7 (1.8-5.6) | 4.9 (2.7-7.8) | 4.4 (2.5-7.0) | 4.6 (2.2-7.1) | **<0.0001** | **<0.0001** | **<0.0001** |
| DHS2 | Haller Index at T6 | 788 | 2.22 (2.02-2.42) | 2.24 (2.06-2.49) | 2.26 (2.06-2.47) | 2.15 (1.98-2.33) | 0.14 | 0.073 | 0.15 |
| (Not in | Haller Index at T8 | 788 | 2.15 (1.99-2.36) | 2.20 (1.99-2.44) | 2.19 (1.97-2.37) | 2.08 (1.98-2.28) | 0.39 | 0.12 | 0.76 |
| DHS1) | Haller Index at Sup. Xiphoid | 788 | 2.14 (1.97-2.33) | 2.22 (1.99-2.43) | 2.18 (1.97-2.36) | 2.08 (1.94-2.22) | 0.18 | **0.046** | 0.40 |
|  | Correction Index at T6 | 788 | 3.2 (1.5-5.7) | 4.8 (2.5-7.7) | 4.6 (2.6-7.4) | 4.9 (1.4-6.6) | **<0.0001** | **<0.0001** | **<0.0001** |
|  | Correction Index at T8 | 788 | 2.6 (0.7-4.8) | 4.3 (1.6-7.8) | 4.5 (2.0-6.5) | 3.9 (0.9-6.4) | **<0.0001** | **<0.0001** | **<0.0001** |
|  | Correction Index at Sup. Xiphoid | 788 | 2.7 (1.0-4.9) | 4.5 (2.0-7.7) | 4.4 (2.1-6.3) | 4.2 (0.9-6.4) | **<0.0001** | **<0.0001** | **<0.0001** |
| DHS2 | Haller Index at T6 | 992 | 2.19 (2.03-2.37) | 2.27 (2.06-2.50) | 2.18 (1.97-2.45) | 2.41 (2.13-2.56) | **<0.001** | **<0.001** | 0.57 |
| (Repeat | Haller Index at T8 | 992 | 2.15 (1.99-2.33) | 2.20 (2.05-2.43) | 2.10 (1.94-2.34) | 2.38 (2.12-2.60) | **<0.001** | **0.002** | 0.084 |
| from | Haller Index at Sup. Xiphoid | 992 | 2.14 (1.98-2.30) | 2.21 (2.02-2.42) | 2.11 (1.90-2.32) | 2.40 (2.12-2.54) | **<0.0001** | **<0.001** | 0.14 |
| DHS1) | Correction Index at T6 | 992 | 3.3 (1.6-5.6) | 4.5 (2.3-6.8) | 5.2 (2.7-7.8) | 3.1 (1.7-5.1) | **<0.0001** | **<0.0001** | **<0.0001** |
|  | Correction Index at T8 | 992 | 2.8 (1.0-5.0) | 4.1 (1.9-7.3) | 3.7 (1.9-6.8) | 4.6 (3.7-7.8) | **<0.0001** | **<0.0001** | **<0.0001** |
|  | Correction Index at Sup. Xiphoid | 992 | 3.0 (1.4-5.2) | 4.6 (1.9-7.5) | 4.6 (2.4-7.0) | 5.0 (2.9-7.2) | **<0.0001** | **<0.0001** | **<0.0001** |

Abbreviations: DHS1, Dallas Heart Study 1; DHS2, Dallas Heart Study 2; Sup., Superior; IQR, interquartile range
