# Supplementary material for: Prevalence of pectus excavatum in an adult population-based cohort estimated from radiographic indices of chest wall shape
Source: PLoS One. 2020 May 7;15(5):e0232575. doi: 10.1371/journal.pone.0232575 (PMC7205298; doi:10.1371/journal.pone.0232575)
Supplement: S5 Table — (DOCX) [file pone.0232575.s005.docx]

**Supplementary Table 5. Linear Models of the Haller and Correction Index of the Dallas Heart Study (DHS1) Cohort (n=2685) at Three Axial Levels (T6, T8 and Superior Xiphoid)**

|  | **Haller Index – T6** | | **Haller Index – T8** | | **Haller Index – Superior Xiphoid** | |
| --- | --- | --- | --- | --- | --- | --- |
| **Haller Index** | **R2 = 0.23** | | **R2 = 0.22** | | **R2 = 0.23** | |
| Factor | **Beta (SE)** | **P-value** | **Beta (SE)** | **P-value** | **Beta (SE)** | **P-value** |
| Ethnicity: Black | ref | - | ref | - | ref | - |
| Ethnicity: White | -0.047 (0.039) | 0.23 | -0.009 (0.04) | 0.82 | -0.009 (0.039) | 0.81 |
| Ethnicity: Hispanic | -0.261 (0.051) | **<0.0001** | -0.323 (0.052) | **<0.0001** | -0.285 (0.052) | **<0.0001** |
| Ethnicity: Other | 0.197 (0.124) | 0.11 | 0.116 (0.125) | 0.35 | 0.103 (0.125) | 0.41 |
| Male Gender | -0.403 (0.047) | **<0.0001** | -0.403 (0.048) | **<0.0001** | -0.408 (0.047) | **<0.0001** |
| Age | -0.025 (0.0002) | **<0.0001** | -0.020 (0.002) | **<0.0001** | -0.0020 (0.002) | **<0.0001** |
| Height | 0.031 (0.003) | **<0.0001** | 0.026 (0.003) | **<0.0001** | 0.029 (0.003) | **<0.0001** |
| Weight | -0.023 (0.001) | **<0.0001** | -0.023 (0.001) | **<0.0001** | -0.024 (0.001) | **<0.0001** |
|  | **Correction Index – T6** | | **Correction Index – T8** | | **Correction Index – Superior Xiphoid** | |
| **Correction Index** | **R2 = 0.073** | | **R2 = 0.14** | | **R2 = 0.12** | |
| Factor | **Beta (SE)** | **P-value** | **Beta (SE)** | **P-value** | **Beta (SE)** | **P-value** |
| Ethnicity: Black | ref | - | ref | - | ref | - |
| Ethnicity: White | 0.241 (0.043) | **<0.0001** | 0.291 (0.042) | **<0.0001** | 0.353 (0.042) | **<0.0001** |
| Ethnicity: Hispanic | 0.383 (0.057) | **<0.0001** | 0.323 (0.054) | **<0.0001** | 0.387 (0.055) | **<0.0001** |
| Ethnicity: Other | 0.094 (0.137) | 0.49 | 0.241 (0.132) | 0.067 | 0.232 (0.133) | 0.081 |
| Male Gender | -0.502 (0.052) | **<0.0001** | -0.539 (0.05) | **<0.0001** | -0.511 (0.051) | **<0.0001** |
| Age | 0.004 (0.002) | **0.048** | 0.003 (0.002) | 0.16 | 0.008 (0.002) | **<0.0001** |
| Height | 0.015 (0.003) | **<0.0001** | 0.026 (0.003) | **<0.0001** | 0.022 (0.003) | **<0.0001** |
| Weight | -0.008 (0.001) | **<0.0001** | -0.017 (0.001) | **<0.0001** | -0.013 (0.001) | **<0.0001** |
