# Supplementary material for: Prevalence of pectus excavatum in an adult population-based cohort estimated from radiographic indices of chest wall shape
Source: PLoS One. 2020 May 7;15(5):e0232575. doi: 10.1371/journal.pone.0232575 (PMC7205298; doi:10.1371/journal.pone.0232575)
Supplement: S6 Table — (DOCX) [file pone.0232575.s006.docx]

**Supplementary Table 6. Linear Models of Chest Wall Shape for the Unrelated Dallas Heart Study (DHS2) Cohort (n=788) at Three Axial Levels (T6, T8 and Superior Xiphoid)**

| **DHS2 (n=788)** | **Haller Index – T6** | | **Haller Index – T8** | | **Haller Index – Superior Xiphoid** | |
| --- | --- | --- | --- | --- | --- | --- |
|  | **R2 = 0.33** | | **R2 = 0.33** | | **R2 = 0.34** | |
|  | **Beta (SE)** | **P-value** | **Beta (SE)** | **P-value** | **Beta (SE)** | **P-value** |
| Ethnicity: Black | ref | - | ref | - | ref | - |
| Ethnicity: White | -0.018 (0.069) | 0.79 | -0.056 (0.069) | 0.42 | -0.025 (0.069) | 0.71 |
| Ethnicity: Hispanic | -0.16 (0.095) | 0.09 | -0.294 (0.095) | **0.0021** | -0.241 (0.095) | **0.011** |
| Ethnicity: Other | -0.41 (0.199) | **0.040** | -0.391 (0.199) | 0.050 | -0.391 (0.198) | **0.049** |
| Male Gender | -0.316 (0.089) | **<0.001** | -0.429 (0.089) | **<0.0001** | -0.383 (0.088) | **<0.0001** |
| Age | -0.024 (0.003) | **<0.0001** | -0.019 (0.003) | **<0.0001** | -0.0019 (0.003) | **<0.0001** |
| Height | 0.022 (0.005) | **<0.0001** | 0.022 (0.005) | **<0.0001** | 0.022 (0.005) | **<0.0001** |
| Weight | -0.021 (0.001) | **<0.0001** | -0.022 (0.001) | **<0.0001** | -0.022 (0.001) | **<0.0001** |
|  | **Correction Index – T6** | | **Correction Index – T8** | | **Correction Index – Superior Xiphoid** | |
|  | **R2 = 0.12** | | **R2 = 0.23** | | **R2 = 0.22** | |
|  | **Beta (SE)** | **P-value** | **Beta (SE)** | **P-value** | **Beta (SE)** | **P-value** |
| Ethnicity: Black | ref | - | ref | - | ref | - |
| Ethnicity: White | 0.335 (0.079) | **<0.0001** | 0.368 (0.074) | **<0.0001** | 0.373 (0.075) | **<0.0001** |
| Ethnicity: Hispanic | 0.331 (0109) | **0.0025** | 0.291 (0.102) | **0.0044** | 0.272 (0.103) | **0.0084** |
| Ethnicity: Other | 0.158 (0.228) | 0.49 | 0.084 (0.214) | 0.70 | 0.084 (0.215) | 0.70 |
| Male Gender | -0.237 (0.102) | **0.020** | -0.477 (0.095) | **<0.0001** | -0.403 (0.096) | **<0.0001** |
| Age | 0.003 (0.003) | 0.24 | 0.006 (0.003) | **0.039** | 0.008 (0.003) | **0.0064** |
| Height | 0.004 (0.005) | 0.44 | 0.019 (0.005) | **<0.001** | 0.014 (0.005) | **0.0047** |
| Weight | -0.010 (0.002) | **<0.0001** | -0.016 (0.001) | **<0.0001** | -0.015 (0.001) | **<0.0001** |
